# Dr. Charles S. Rudolph Reed

**Published:** 1914-12

**Authors:** 


**Dr. Charles S. Rudolph Reed,** P. & S. 1879, died in St. Elizabeth's Hospital, Utica, following an operation, recently, aged 62. He was Consulting Surgeon in Diseases of the Eye, Ear, Nose and Throat to that institution.

